# Advancing Alzheimer’s Disease Care and Services Among Racial and Ethnic Minorities

**DOI:** 10.1093/geroni/igaa057.2682

**Published:** 2020-12-16

**Authors:** Lenora Smith, Roland Thorpe

## Abstract

Research shows consistent and adverse disparities among racial and ethnic minorities compared to non-Hispanic Whites in the prevalence and incidence of Alzheimer’s disease, mortality, participation in clinical trials, use of medications and other interventions, health care expenditures, and quality-of-life outcomes. The literature suggests numerous underlying causes, including factors related to measurement of the disease, genetics, socioeconomic factors, cultural differences, lack of culturally competent interventions, and discrimination in services and care. Although these disparities are well known, little is known about the effectiveness of various strategies to address these differences within the context of Alzheimer’s disease services and care. This symposium aims to contribute to this knowledge. The first presentation examines the role of race with marital status and risk for dementia using data from the Health and Retirement Study. Results suggest differences for unmarried White and unmarried older adults of color, which can inform dementia care services. The second presentation highlights the opportunities and challenges of facilitating cognitive impairment screenings among African American congregations. The third presentation introduces attitudes about brain donation among African American research participants and suggestions to increase involvement. The symposium concludes with a presentation on hearing care disparities in dementia with practical recommendations on how to close this gap in hearing care. The findings from these papers contribute significantly to the impact of ethnoracial differences in dementia and the need to include more diverse populations in ADRD research to promote equity. Alzheimer’s Disease Research Interest Group Sponsored Symposium.

